# Degenerative and Regenerative Actin Cytoskeleton Rearrangements, Cell Death, and Paradoxical Proliferation in the Gills of Pearl Gourami (*Trichogaster leerii*) Exposed to Suspended Soot Microparticles

**DOI:** 10.3390/ijms242015146

**Published:** 2023-10-13

**Authors:** Nikolay P. Sudakov, Hung-Ming Chang, Ting-Yi Renn, Igor V. Klimenkov

**Affiliations:** 1 Department of Cell Ultrastructure, Limnological Institute, Siberian Branch, Russian Academy of Sciences, 3 Ulan-Batorskaya St., 664033 Irkutsk, Russia; npsudakov@gmail.com; 2 Department of Anatomy and Cell Biology, School of Medicine, College of Medicine, Taipei Medical University, Taipei 110301, Taiwan; taiwanose@tmu.edu.tw; 3 Graduate School of Biomedical and Health Sciences, Hiroshima University, Hiroshima 734-8553, Japan; littlenorenn@gmail.com

**Keywords:** soot microparticles, fish, gills, F-actin, apoptosis, proliferation, bioindication

## Abstract

The effect is studied of water-suspended soot microparticles on the actin cytoskeleton, apoptosis, and proliferation in the gill epithelium of pearl gourami. To this end, the fish are kept in aquariums with 0.005 g/L of soot for 5 and 14 days. Laser confocal microscopy is used to find that at the analyzed times of exposure to the pollutant zones appear in the gill epithelium, where the actin framework of adhesion belts dissociates and F-actin either forms clumps or concentrates perinuclearly. It is shown that the exposure to soot microparticles enhances apoptosis. On day 5, suppression of the proliferation of cells occurs, but the proliferation increases to the control values on day 14. Such a paradoxical increase in proliferation may be a compensatory process, maintaining the necessary level of gill function under the exposure to toxic soot. This process may occur until the gills’ recovery reserve is exhausted. In general, soot microparticles cause profound changes in the actin cytoskeleton in gill cells, greatly enhance cell death, and influence cell proliferation as described. Together, these processes may cause gill dysfunction and affect the viability of fish.

## 1. Introduction

Soot is one of the most common environmental pollutants [1,2]. Soot microparticles emerge during smoldering combustion of biomass and fossil fuel [3]. The microparticles initially get into the atmosphere and subsequently settle on land and water surfaces [2,4]. In aquatic environments, soluble forms of soot engage in circulation and settling processes and can persist for tens of thousands of years in bottom sediments [5,6]. The structure and chemical composition of soot microparticles determines their toxic effect on biological objects. Soot microparticles are one of the sources of polycyclic aromatic hydrocarbons (PAHs) in aquatic environments, i.e., a ubiquitous pollutant in fresh and marine water bodies, which has been affecting aquatic organisms ever more intensively with the expansion of human activity [7]. It was found that the products of PAH metabolism can modify biomolecules by forming adducts with proteins, nucleic acids, and lipids [8,9,10,11,12]. A toxic effect is also observed for soot-associated metals and metalloids either in their pure forms or as part of various compounds [13,14,15,16,17]. Apart from the extractable components, the harmful properties of soot also depend on the size [18] and structure [19] of its microparticles. The effect of soot on the inhabitants of aquatic ecosystems has been studied to a lesser extent [20,21] than for terrestrial organisms [22,23,24]. The study of the effect of soot on hydrobionts is relevant for developing bioindication technologies for this pollutant, for assessing the environmental risks in terms of biodiversity conservation, and for addressing issues of fundamental toxicology and environmental medicine. The work focusing on the effect of soot and its components on hydrobionts includes studies on crustaceans [25] and mollusks [20]. The effect of PAHs on fish embryogenesis was studied [26,27,28], including the mechanisms underlying the formation of heart chambers in zebrafish (*Danio rerio*) embryos [21]. Fish are the most appropriate object for assessing the state of aquatic ecosystems because of their universal distribution and key role in food chains [29]. Fish gills are one of the most promising organs for bioindication of water pollution [30,31] due to their direct contact with the environment [32], which makes gills one of the main routes for pollutants to enter the body [33]. In addition to the gas exchange function, gills also participate in osmoregulation, acid–base balance control, nitrogen metabolism product excretion [34], and xenobiotics metabolism and excretion [35,36]. The presence of chemoreceptor cells in these fish organs [37,38] predetermines the involvement of gills in regulating the activity of the respiratory and cardiovascular systems. All these important functions underlie the high sensitivity of gills to pollution and other environmental changes. Functional disturbances in gills can be critical for life activity in fish, which is why it is important to monitor these organs for the conservation of water bodies’ biodiversity.

Our previous works were devoted to the effects of short-term (5 days) exposure to soot on Baikalian fishes, evolutionary adapted to the clearest fresh water of Lake Baikal [39]. The data of these experiments provided evidence of a decrease in mitochondrial activity, an increase in reactive oxygen species production, and an increase in the frequency of programmed cell death in the gill epithelium, and macroautophagy in chloride cells of *Paracottus knerii* (Dybowski, 1874) gills under the influence of soot. The obtained data highlighted the necessity of the further study of the impacts of soot on fish with higher tolerances to changes in the environment. Therefore, we chose pearl gourami, *Trichogaster leerii* (Bleeker, 1852), used in toxicological experiments [40], as well as other representatives of genus Trichogaster [41,42]. The presence of a labyrinth organ allows these fish to also breathe with air, decreasing the loading on the respiratory function of the gills [43,44]. This aspect decreases the possible effects of hypoxia that can mask or imitate the toxic effects of soot on fish gills during experiments modeling water polluted with soot [45].

Thus, the aim of study is the analysis of the influence of soot microparticles on intracellular actin cytoskeleton structural rearrangements, apoptosis, and cell proliferation activity in the gills of *Trichogaster leerii*. These processes may impair gill function in the fish and reduce their ability to adapt to environmental changes.

## 2. Results and Discussion

### 2.1. Degenerative and Adaptive Rearrangements of Actin Microfilaments in Gill Epithelium under the Influence of Soot

**Figure 1:**
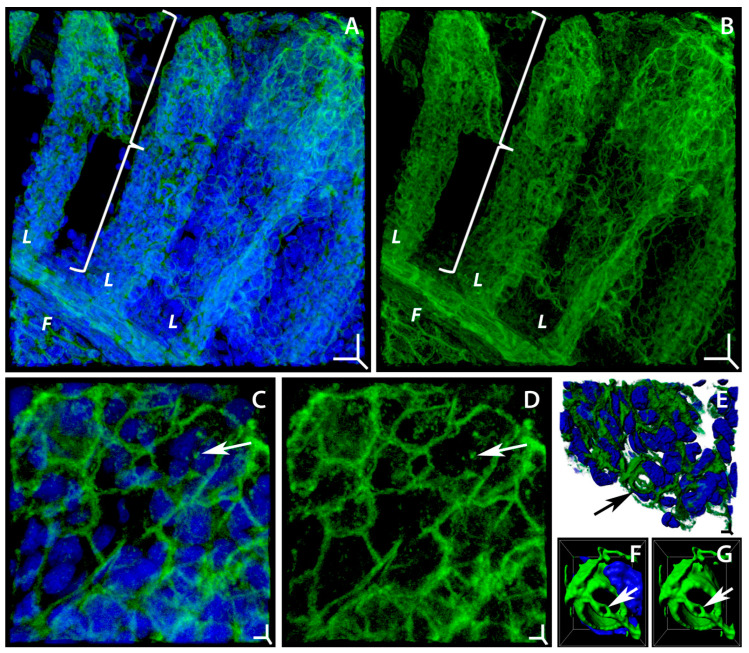
Structural organization of actin microfilaments in gill cells of *Trichogaster leerii* in control. Laser scanning confocal microscopy, staining of actin microfilaments (Alexa Fluor 488 phalloidin, green) and cell nuclei (Hoechst 33342, blue). (**A**,**B**) Fragment of gill filament (*F*) with lamellae (*L*, brace) that has a meshy structure formed with actin-adhesive circumferential belts of epithelial cells contacting with one another ((**A**) merged; (**B**) F-actin). (**C**,**D**) Fragment of gill lamella surface with irregular structure of actin-adhesive circumferential belts; actin patches indicated with arrow ((**C**) merged; (**D**) F-actin). (**E**) Surface of gill epithelium with mucous cell (arrow); (**F**,**G**) mucous cell of gill epithelium, on the apical side of cell arrow shows actin ring fixed with two actin cables ((**F**) merged, (**G**) F-actin). Three-dimensional volume rendering mode: (**A**–**D**) maximum intensity projection (MIP); (**E**) normal shading; (**F**,**G**) surface rendering. Three-dimensional scale bars (XYZ); (**A**,**B**) 10 µm; (**C**–**E**) 1 µm. Box (XYZ); (**F**,**G**) 7, 8.5, 5 µm.

Laser confocal microscopy data indicate that cellular elements of the gill epithelium in *T. leerii* belonging to the control group have circumferential adhesion belts composed of F-actin, which form a polygonal structure along the entire contour of the apical region of the cell (Figure 1A–D). The individual sides of these belts are of different lengths (from 1.3 to 8.7 µm, on average 3.8 ± 1.4 µm) and form 5–7 angles. The width of the actin belts is 0.68 ± 0.22 µm, while the diameter of the respiratory cell compartment varies in the range 2.3–9.4 µm, averaging 5.1 ± 1.4 µm. These actin structures in neighboring cells are connected by intercellular adhesion molecules into a single network covering the entire area of the gill lamellae. The identified structure of the gill cytoskeleton is consistent with the data obtained by Sandbichler et al. [46] on rainbow trout (*Oncorhynchus mykiss*) gills. In general, this organizational principle is typical of all epithelial cells that form the integument of various tissues. Epithelia were found to have two important types of intercellular contacts associated with the actin cytoskeleton: tight and adherens junctions [47]. These contact types are also present in the gill epithelium [46]. It was found that tight and adherens junctions firmly bind the apical poles of cells [48]. The molecular composition of tight (*zonulae occludentes*) and adherens junctions (*zonulae adherentes*) was detailed by Steinke A. et al. [49]. The basis of adherens junctions is formed by E-cadherin [50]. In general, the observed structure of the gill epithelium actin network is adapted to maintain the mechanical integrity of this tissue under constant contact with the water flow and to ensure the functions performed by the gills.

The epithelial cells of the gills, mainly in the marginal zone, contain elongated ellipsoidal and spheroidal dense F-actin structures with a diameter of 0.27 to 1.81 µm, averaging 0.79 ± 0.30 µm (Figure 1C,D). The number of these structures per cell varies from 0 to 26, averaging 7.0 ± 5.0. These actin structures are referred to as actin patches [51]. They are ubiquitous for eukaryotic cells as they have been described in a fairly wide range of eukaryotes [52,53,54,55]. The basis for the formation and maintenance of actin patches is the Arp2/3 complex [52]. Actin patches have a complex protein composition, which varies depending on the patch type [56,57]. These structures are very dynamic [58] and capable of rapid movement within the cell [51]. The patches have been shown to play a role in endocytosis [52]; in regulating the selective direction of vesicular transport to the axon or dendrites [58] and organelle transport [59]; and in the cell response to mechanical impacts, to the extracellular matrix structure [55], and to the plasma membrane curvature [60]. It can be assumed that the actin patches in the pavement cells of the gill epithelium are associated with similar processes, which are necessary for the adequate functioning of these cells.

The 3D structure of the F-actin network in the gill epithelium was analyzed to reveal secretory (mucous) cells. Their actin cytoskeleton is represented by a barrel-shaped solid structure along the entire lateral contour of the cell with a diameter of 5.02 ± 0.96 µm, with a rim attached to two parallel actin strands in the apical region with a central pore (Figure 1E–G; Supplementary Video S1) of 0.54 ± 0.12 µm in diameter. This observation is consistent with the organizational principles of the mucous cells of the conjunctival epithelium [61], but the rim structure at the center of the apical region of the secretory cell has not been described as of yet. It can be assumed to participate in compartmentalizing the cell contents and to be essential for the regulation of mucus secretion.

**Figure 2:**
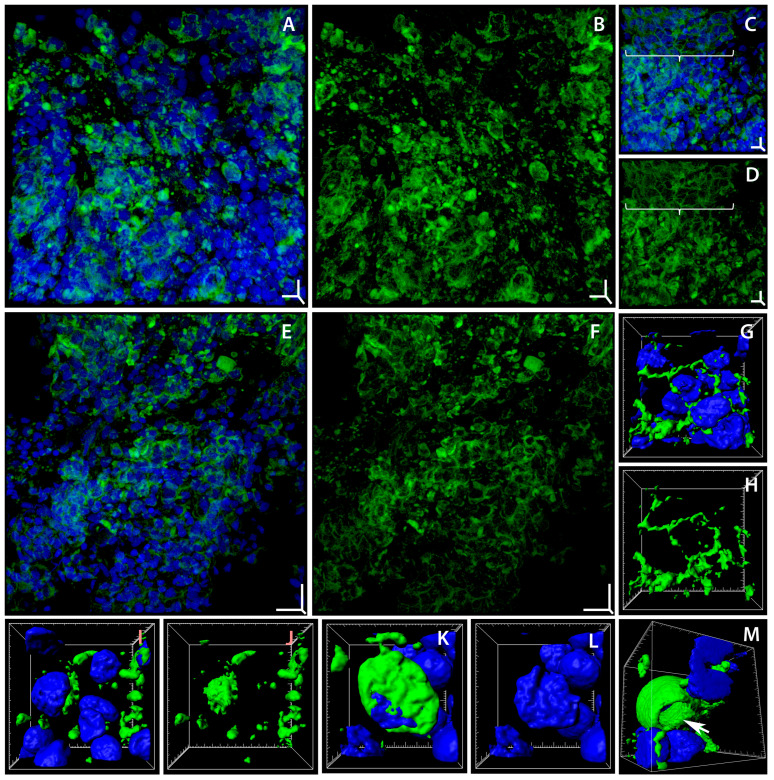
Degenerative changes in actin microfilaments in gill cells of *Trichogaster leerii* exposed to soot microparticles. Laser scanning confocal microscopy, staining of actin microfilaments (Alexa Fluor 488 phalloidin, green) and cell nuclei (Hoechst 33342, blue). (**A**,**B**) Area of degenerative changes in actin microfilaments in gill cells; day 5 of exposure to soot microparticles ((**A**) merged; (**B**) F-actin). (**C**,**D**) Fragment of gill epithelium with area of F-actin degenerative changes neighboring zone of epithelium with unchanged structure of actin microfilaments (brace); day 14 of soot exposure ((**C**) merged; (**D**) F-actin). (**E**,**F**) Area of degenerative changes in actin cytoskeleton of gill epithelium; 14 days of soot microparticle influence ((**E**) merged; (**F**) F-actin). (**G**,**H**) Fragment of epithelium with partial dissociation of actin-adhesive circumferential belts ((**G**) merged; (**H**) F-actin). (**I**,**J**) Cells with full dissociation of actin-adhesive circumferential belts, with aggregation to small and large clumps from F-actin ((**I**) merged; (**J**) F-actin). (**K**,**L**) Cell with F-actin continuous perinuclear zone ((**K**) merged; (**L**) nuclei). (**M**) Mucous cell lost F-actin rim on the apical side, indicated with arrow (merged). Three-dimensional volume rendering mode: (**A**–**F**) maximum intensity projection (MIP); (**G**–**M**) surface rendering. Three-dimensional scale bars (XYZ): (**A**–**D**) 5 µm; (**E**,**F**) 10, 10, 4 µm. Box (XYZ): (**G**,**H**) 14, 13.3, 7 µm; (**I**,**J**) 13.1, 13, 7 µm; (**K**,**L**) 8.5, 9, 5 µm; (**M**) 12.5, 12.5, 12.5 µm.

It was found that degenerative changes occured in the actin cytoskeleton of *T. leerii* gills on days 5 and 14 following the exposure to soot microparticles (Figure 2A–F). Epithelium zones appear where the actin framework of the adhesion belts thins out, fragments, and dissociates (Figure 2G,H), while the F-actin of these cells either redistributes into aggregates or clumps of 1.37 ± 0.55 µm in size (Figure 2I,J), or forms a continuous perinuclear zone (Figure 2K,L). Actin patches survive in cells uninvolved in the changes described above, but they disappear during the degenerative rearrangements of F-actin. Meanwhile, the above-described rearrangements of actin microfilaments do not occur in single cells but embrace tissue zones adjacent to epithelium regions characterized by the normal structure of the actin cytoskeleton (Figure 2C,D). These zones with a disrupted structure of the actin cytoskeleton occupy 30% of the area of the gill epithelium.

Data from various experiments indicate that oxidative stress induced by various factors can promote actin depolymerization [62], actin network fragmentation [63,64], and the formation of atypical F-actin aggregates [64,65]. The available information on the effect of soot microparticles on the actin cytoskeleton is scarce and contradictory. Thus, evidence was obtained for the absence of an effect of soot on the structure of F-actin in the various tissues of *Drosophila melanogaster* larvae [22]. On the other hand, a short-term exposure of an endotheliocyte culture to soot aggregates was found to disrupt the F-actin distribution in cells [66]. The disorganization and rearrangements in the system of actin microfilaments in cells are caused by carbon nanotubes [67]. Evidence was obtained of actin cytoskeleton rearrangements accompanied by the formation of scattered intracellular aggregates under the influence of benzo[*a*]pyrene contained in soot [68]. It was found that benzo[*a*]pyrene and dibenzo[*a,l*]pyrene change the expression of the genes of cytoskeletal components [69].

The possible mechanisms underlying the observed rearrangements of actin microfilaments have numerous aspects due to the complexity of the structural and functional organization of the cytoskeleton. It was shown that the actin cytoskeleton undergoes reorganization under oxidative stress due to the activation of mitogen-activated protein (MAP) kinase stress-activated protein kinase-2/p38 (SAPK2/p38), which leads, through the MAP kinase-activated protein kinase-2/3, to the phosphorylation of HSP27, which is a modulator of actin polymerization [70,71]. A possible mechanism for PAHs to induce F-actin rearrangements is to act via the aryl hydrocarbon receptor (AhR) [72], which activates the focal adhesion kinase through Src induction [73], which leads to the activation of the focal adhesion complex. It has been shown that actin is one of the main objects of carboxylation and glutathionylation under oxidative stress [74], which leads to its rearrangements, including aggregation and concentration in the perinuclear space [75,76]. The development of oxidative stress in fish gill cells when exposed to soot was shown in a similar experiment in our previous study [39]. This suggests that the formation of actin aggregates in gill epithelial cells after 5 and 14 days of exposure to soot microparticles is a consequence of oxidative stress.

Since actin microfilaments play an important role in cells, disruptions in their structure are crucial for the functioning of gill epithelial cells. It has been shown that actin depolymerization leads to the weakening of intercellular contacts, to the impairment of the cells’ natural shape, and to the loss of tissue stability [77,78]. One of the consequences resulting from the oxidative modification of actin is the intensification of cell death [79]. Actin cytoskeleton rearrangements are a critical event in the implementation and modulation of apoptosis [80,81,82]. Disruptions in the normal structure of F-actin in the cell were also shown to play an active role in the development of apoptosis [83].

We showed that on days 5 and 14, the actin skeletons of mucous cells became deformed, with the loss of the fine structure of the apical region (Figure 2M; Supplementary Video S2), which may be due to hypersecretion. The activation of mucus hypersecretion by gill secretory cells in fish when exposed to soot microparticles was revealed by us previously [39].

**Figure 3:**
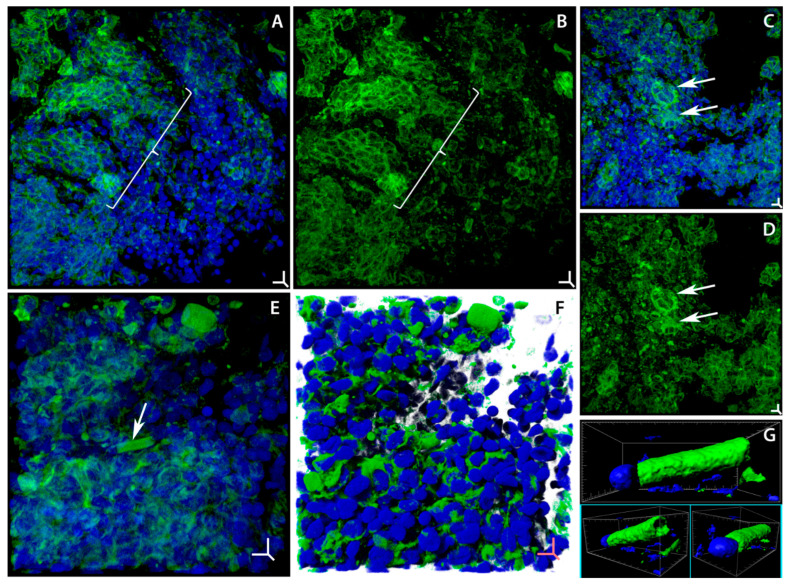
Adaptive rearrangements of actin microfilaments in gill cells of *Trichogaster leerii* under the influence of soot microparticles. Laser scanning confocal microscopy, staining of actin microfilaments (Alexa Fluor 488 phalloidin, green) and cell nuclei (Hoechst 33342, blue). (**A**,**B**) Fragment of gill epithelium with degenerative changes in actin cytoskeleton contains large area of cells with thickened actin-adhesive circumferential belts; pointed with brace ((**A**) merged; (**B**) F-actin). (**C**,**D**) Group of cells with thickened actin-adhesive circumferential belts (arrows) is in the area of epithelium with degenerative changes in F-actin ((**C**) merged; (**D**) F-actin). (**E**,**F**) Fragment of epithelium with degenerative changes in actin microfilaments, with newly formed (young) cell (arrow) migrating into lesioned area of tissue. (**G**) Detailed image (in different projections) of newly formed migrating cell with special torpedo-shaped actin “sheath”, which is essential for movement through epithelial tissue (merged). Three-dimensional volume rendering mode: (**A**–**E**) maximum intensity projection (MIP); (**F**) shadow projection; (**G**) surface rendering. Three-dimensional scale bars (XYZ): (**A**–**D**) 4.5 µm; (**E**,**F**) 5 µm. Box (XYZ): (**G**) 13.5, 5.5, 7 µm.

Along with the areas characterized by degenerative changes in the F-actin network, the gill epithelium of the experimental fish has groups of cells with actin belts that are thicker than in the controls, located adjacent to the zones of toxic tissue damage (Figure 3A–D). These changes appear to be aimed at increasing the mechanical strength and maintaining the integrity of the gill epithelium, which experiences local weakening of the actin network as a result of the cytotoxic effect of soot microparticles. Such reactions of the actin cytoskeleton, which are aimed at adapting to mechanical stress in tissues, are universal [84].

We revealed yet another adaptive change in the structure of the actin cytoskeleton of gill epithelial cells, i.e., torpedo-shaped actin “sheaths” at the leading end of migrating cells, which we recorded on day 14 of soot exposure in 10% of the Z-stacks of gill filaments (Figure 3E–G). We first discovered such structures in the olfactory epithelium of *Cottocomephorus inermis* during active migration of newly formed neurons through dense tissue layers [85]. This kind of structure has not been recorded previously in gill epithelium. We believe that the young migrating cells are designed to repair damaged tissue areas. Figure 3E,F shows a cell with an actin sheath that is moving towards a tissue area with a large group of cells with degenerative changes in actin microfilaments. In general, the actin cytoskeleton of the gill epithelium is very sensitive to the effects produced by the components of soot microparticles, which cause a complex of degenerative and adaptive changes in the cells; the ratio of these processes determines the structural integrity and functionality of gills in fish.

### 2.2. Programmed Cell Death in Gill Epithelium under the Influence of Soot

**Figure 4:**
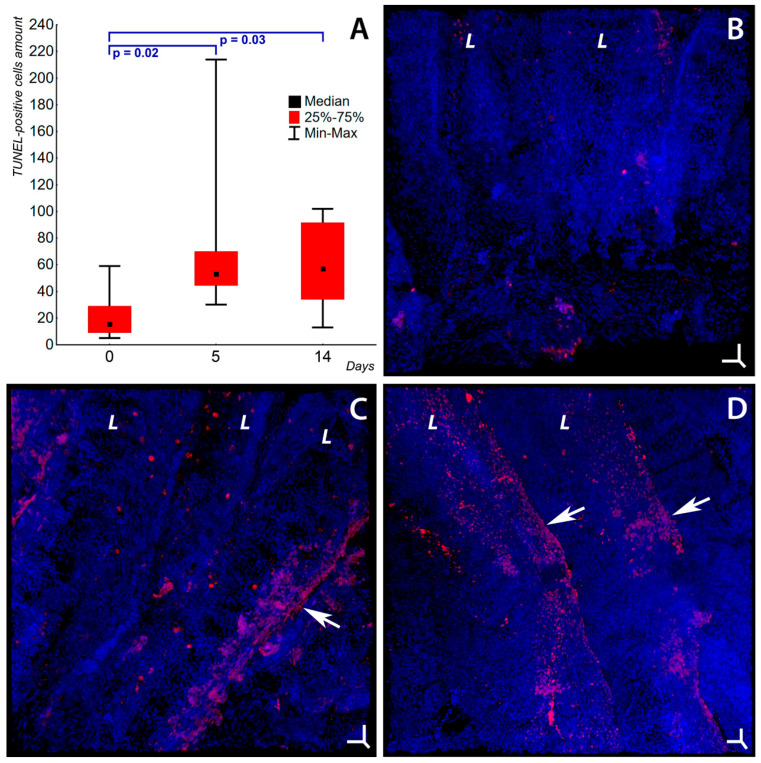
Apoptosis activity in gills of *Trichogaster leerii* in control and under the soot microparticles’ influence. Laser scanning confocal microscopy, staining of cell nuclei with fragmented DNA (TUNEL, stain—Alexa Fluor 594, red) and cell nuclei (Hoechst 33342, blue). (**A**) Box plot of apoptosis activity in gills in control (1), on days 5 (2) and 14 (3) of soot exposure (*x*-axis); *y*-axis shows TUNEL-positive cells per 1 × 10^6^ µm^3^ tissue volume, obtained by the quantitative analysis of the Z-stacks (confocal microscopy). Box plot shows natural apoptosis level in control (0 days), and increase in apoptotic activity on days 5 and 14 of incubation with soot. (**B**) Natural activity of apoptosis and peculiarities of TUNEL-positive cells’ localization in gill lamellae (*L*) in control group. (**C**,**D**) The increase in apoptosis activity on days 5 and 14 of soot exposure, respectively. Predominant localization of TUNEL-positive cells at the edges of gill lamellae (*L*), most affected by mechanical action of water flow (direction indicated with arrow) carrying soot microparticles. (**B**–**D**) Maximum intensity projection 3D volume rendering mode. Three-dimensional scale bars (XYZ): (**B**–**D**) 20 µm.

An analysis of the number of TUNEL-positive cells (Supplementary Figure S1 for positive and negative control) in the gill epithelium of *T. leerii* in the control group revealed active apoptotic processes associated with the natural self-renewal of tissue (Figure 4A,B, Supplementary Figure S2A–C). Continuous death of senescent and damaged cells in combination with the division and subsequent differentiation of stem epithelial cells represents an integral feature of all epithelia [86,87,88]. The intensity of this process was virtually no different from the same parameter in *Paracottus knerii*, which we estimated previously in [39]. This fact suggests that this level of activity may be typical of most fish. It was found that TUNEL-positive cells in the gill epithelium of the control group occur both as singly located and as groups, possibly associated with the natural damage to the epithelium as a result of a hydrodynamic force impact. Exposure to soot microparticles on days 5 and 14 of the experiment increased apoptosis in *T. leerii* gills by a factor of 5 (*p* ≤ 0.05) (Figure 4A,C,D, Supplementary Figure S2D–I). When exposed to soot, the main quantity of TUNEL-positive cells in the gill epithelium concentrates at the edges of gill lamellae and filaments, which appear to be the hydrodynamic force impact (shear stress) zones most susceptible to mechanical stress and turbulent fluid flow, which creates optimal conditions for the adhesion of soot microparticles to the tissue surface. The value of this parameter and the localization of apoptotic cells on day 5 of the soot exposure coincide with the data obtained for *P. knerii* with a similar exposure [39]. This is evidence for the universal effect of the soot microparticles used in the experiment on the gill epithelium of fish and for the objectivity of the TUNEL method used to assess the cytotoxic effect of soot microparticles. These facts are important for the formulation of a unified concept for the toxic effect of soot on fish gills and for the development of bioindication technologies for these contaminants. Possible mechanisms of apoptosis induction by soot microparticles in fish gills can be reconstructed using the data obtained mainly on terrestrial vertebrates. An activation of programmed cell death by soot microparticles has been shown for myocardial cells in zebrafish [89]; liver cells in mice [90]; lungs [91], hypothalamus, and testicles in rats [92]; corneal epithelial cells [93], and human lung epithelial cells (A549) [94]. Soot-induced cell apoptosis is associated with the development of oxidative stress [90,92]. This process is due to the production of the superoxide anion with the parallel weakening of antioxidant protection, which manifests itself in the decreased activity of glutathione and superoxide dismutase [95], glutathione peroxidase, and catalase [96]. This is accompanied by a decrease in the mitochondrial potential, an activation of Bax, and a release of cytochrome c from mitochondria [97]. Another possible mechanism for apoptosis induction under soot-induced oxidative stress is associated with disruptions of the Ca^2+^ content in the endoplasmic reticulum and mitochondria [98]. Several studies have shown that oxidative stress during these processes can lead to a violation in the integrity of cell membranes [14,15]. It was also shown that soot nanoparticles cause metabolic disorders associated with the Krebs cycle and alanine, aspartate, and glutamate metabolism. The violation of energy metabolism processes and the disruption of vital signaling pathways ultimately lead to the induction of apoptosis [99]. Soot microparticles were also found to exert a cytotoxic effect on corneal epithelial cells, which is accompanied by the activation of the NLRP3 inflammosome [93], which is involved in the development of pyroptosis [100]. The toxic effect of soot microparticles on human lung epithelial A549 cells due to oxidative stress with subsequent autophagy and cell death was described in [94]. There is a multitude of factors by which soot microparticles can induce programmed cell death. A pronounced proapoptotic effect is known for soot PAHs: fluorene and phenanthrene [101], benz[*a*]pyrene [102,103,104,105], dibenzo[*a,l*]pyrene [106], 7,12-dimethylbenz[*a*]anthracene [104,107], 1-methylpyrene and perylene [108], fluoranthene [109], and 3-nitrofluoranthene [110]. Other soot components that can cause a cytotoxic effect are the metals associated with soot microparticles (Mn, Al) and silica, which we identified previously [39]. A cytotoxic effect was shown for Mn [13], nanoparticles with Al_2_O_3_ [111], and for amorphous Si [112]. The cytotoxic effect of soot microparticles was also shown to increase with the increase in their size [18] and concentration [113]. A dose-dependent effect is typical both of conventional and of ozonized [114] and nanostructured soot [98]. The composition of soot is also important for the induction of apoptosis, since ozonized [115] and more graphitized [19] soot contributes to a greater degree to the increased intensity in cell death processes.

Thus, on days 5 and 14, soot causes programmed death of epithelial cells in *T. leerii* gills. Possible mechanisms of this process could be as follows: the induction of oxidative stress, the activation of proapoptotic mitochondrial mechanisms, and the release of Ca^2+^ into the cytosol. The activation of programmed cell death in fish gills under the influence of soot in the experiment indicates the sensitivity of this indicator and its high potential for assessing the degree of impact of this pollutant on hydrobionts.

### 2.3. Paradoxical Effect of Soot Microparticles on Cell Proliferation in Fish Gill Epithelium

**Figure 5:**
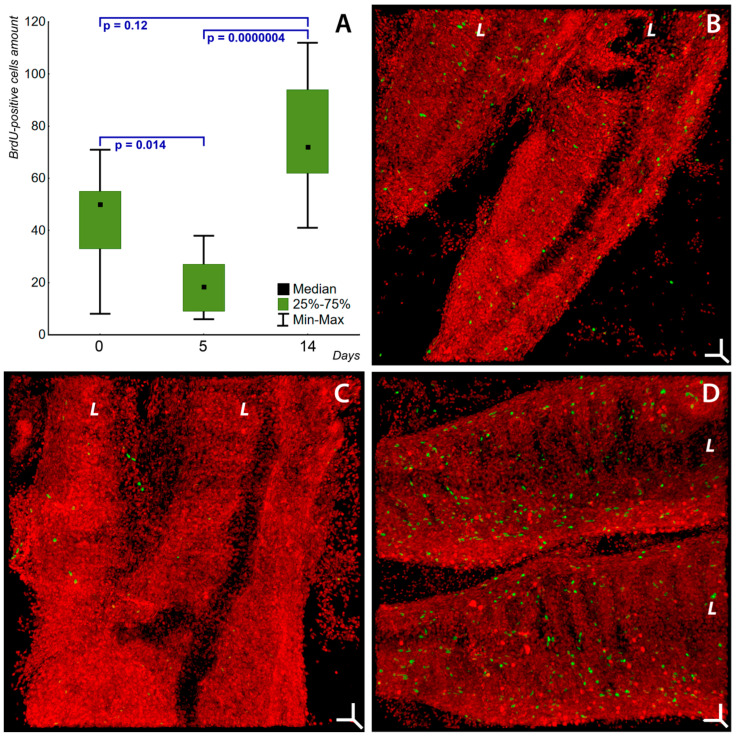
Cell proliferation in gills of *Trichogaster leerii* in the control and under the impact of soot microparticles. Laser scanning confocal microscopy, staining of cell nuclei with incorporated BrdU in DNA (FITC-conjugated anti-BrdU antibodies, green) and cell nuclei (7-AAD, red). (**A**) Box plot of proliferative activity in gills in control (0 days), and on days 5 and 14 of soot exposure (*x*-axis); *y*-axis shows BrdU-positive cells per 1 × 10^6^ µm^3^ tissue volume, obtained by the quantitative analysis of the Z-stacks (confocal microscopy). Box plot shows decrease in cell proliferation intensity in gills on day 5 (2) of exposure to soot, and cell proliferation increase on day 14 (3), to values close to the control. (**B**) Peculiarities of natural proliferative activity of gill lamellae (*L*) cells in control group. (**C**) Decrease in cell proliferation in gill lamellae (*L*) on day 5 of soot microparticle exposure. (**D**) Increase in cell proliferation in gill lamellae (*L*) on day 14 of soot microparticle exposure. (**B**–**D**) Maximum intensity projection 3D volume rendering mode. Three-dimensional scale bars (XYZ): (**B**–**D**) 20 µm.

An analysis of the proliferative activity in the gill cells of *T. lerii* in the control group showed the amount of BrdU-positive cells (Figure 5A,B, Supplementary Figure S3A–C) that corresponds to the natural level of proliferation processes in the gill epithelium. As we mentioned in the previous section, these processes, along with programmed cell death, constitute together a continuous process of self-renewal, which is typically observed to proceed at high rates in epithelial tissues [88,116]. The data obtained are consistent with the results on the natural background of proliferation in *T. leeri* gills [117], but since the studies employed different methodological approaches (analysis of thin sections vs. 3D analysis of Z-stacks), it is very difficult to compare the quantitative data. The level of proliferative processes in fish gills during their regeneration was well studied in *Danio rerio* after tissue resection [118,119]. The similarities of the regeneration processes in fish gills and mammal (including human) lungs were discussed in [120]. We found that the presence of soot microparticles suppresses proliferation on day 5 by a factor of 3 (*p* = 0.014) in comparison with the control (Figure 5A,C, Supplementary Figure S3D–F). However, on day 14, the soot-exposed fish demonstrate a paradoxical increase in this process (Figure 5A,D, Supplementary Figure S3G–I): in comparison with day 5 of soot exposure, this indicator increases by a factor of 4 (*p* = 0.0000004), without being noticeably different from the control data (*p* = 0.12). In the control group and after the 5- and 14-day soot incubation, the distribution of BrdU-positive cells was diffuse throughout the entire volume of the gill epithelium, and this tissue had no areas with reduced or increased proliferative activity in comparison with other tissue zones. Of interest is the simultaneous activation of proliferation on day 14 of soot exposure and the appearance of signs of active migration in newly formed cells (torpedo-shaped actin sheaths) into the damaged tissue zones. The coincidence of these two events is an objective sign of compensatory activation of regenerative processes in the tissue that has received toxic damage. Such a paradoxical effect of soot microparticles on the proliferation of gill cells can be explained by summarizing the experimental data on the effect of soot on cell proliferation. On the one hand, soot has been shown to cause reduced proliferative activity of the lung epithelial cell line A549, which is associated with increased expression of anti-proliferative proteins LMNA and PA2G4 [121,122], and also inhibits the proliferation of lung fibroblasts in mice due to cell cycle arrest in the S phase [14]. On the other hand, the proliferation of lung cells in mice, A549 cells [123], and EA.hy926 endothelial cells [124] was shown to activate under the influence of soot. However, in rat lungs, soot microparticles showed different effects on the proliferative activity of different parts of the bronchial and alveolar tree—the respiratory bronchioles had a low value of this indicator while the intensity of cell division in the terminal bronchioles and the main lung parenchyma remained unchanged [125]. Such a paradoxical effect of soot microparticles on proliferation in the gills can be partially explained by the multicomponent composition of these particles, which determines the variety of soot-induced cellular effects. The suppression of proliferation may be due to the previously discussed cytotoxic mechanisms: damage to the actin cytoskeleton and activation of proapoptotic mechanisms. The increased proliferation in the gills under the influence of soot could be caused by the PAHs (despite their proapoptotic activity) that are present in its composition. Such an effect was shown for benzofluoranthenes, benz[*a*]anthracene, and chrysene due to the activation of the Ah receptor [126]. The Ah-receptor-mediated effects are fairly broad and include detoxification, cell proliferation, and migration [127]. However, benz[*a*]pyrene was shown to activate cell proliferation [124] via an Ah-receptor-independent pathway [128]. In addition to the activation of the proliferation of gill epithelial cells by soot components, this process can also be induced by apoptosis (the so-called apoptosis-induced compensatory proliferation) [129]. There is evidence of tissue proliferation being stimulated through the release of IL-6, IL-8, MCP-1, and MMP-9 by neighboring damaged cells [130]. Cell proliferation [131] and migration [132] were also shown to activate in response to chemical tissue damage. This compensatory or chemically induced activation of tissue self-renewal is limited by its regenerative reserves [133]. These regularities were also determined for gills [134]. When the regenerative and differentiation potential of the tissue reserves is exhausted, the tissue function is irreversibly lost [135,136,137], which contributes to a decrease in the viability of the organism or reduces the quality of its life.

Summarizing the above, soot microparticles have a paradoxical effect on the proliferation of gill epithelial cells in fish: on day 5 of exposure, they suppress this process, but on day 14, the proliferation is activated to rates approaching the control values. The primary suppression of the proliferation might be attributed to the pronounced toxic effect of soot microparticles, and its subsequent activation might be due both to the stimulation of Ah-receptor-mediated and -independent signaling pathways by some PAHs and to a compensatory regenerative tissue response to cell damage and death caused by the intoxication. The revealed patterns of the regenerative processes in gills during soot exposure are important for fundamental toxicology and ecological medicine.

## 3. Material and Methods

### 3.1. Animals and Design of Experiment

In the experiment, we used males of the pearl gourami, *Trichogaster leerii* (Bleeker, 1852). The pearl gourami of the control group (group 1, *n* = 10) were kept under standard conditions [117]. A description of the soot microparticles (5–50 nm in size; for elemental composition and PAH content see Supplementary Table S1) used in our experiment was published by Sudakov et al. [39]. In contrast to the control group, a stock solution of soot microparticles was added to the aquarium with the animals of the experimental group (groups 2 and 3; *n* = 10 in each group) in order to obtain a final concentration of 0.005 g/L. This soot solution was prepared according to the method described by Li et al. [138]. We used a concentration of soot in water of 0.005 g/L, as in earlier research on the effects of soot microparticle exposure on fish gills [39]. This water-dissolved soot amount corresponds to the maximally registered soot level in a natural water environment (0.0037 g/L in the Gulf of Mexico) [139]. The animals were removed from the experiment to study the structure of the actin cytoskeleton and the intensity of apoptosis and proliferation in the gills on days 5 (group 2) and 14 (group 3) of the experiment. We used the period of 5 days as in the previous experiment [39] for a comparative study of the short-term impact of the soot microparticles, and 14 days to estimate more delayed effects of soot exposure. To this end, we used tricaine mesylate (MS222) [140] in conformity with the principles of the AVMA Guidelines for the Euthanasia of Animals (2020). Extraction and fixation of the gills during autopsy was performed according to the previously described technique [39]. All the manipulations with the experimental fish were approved by the Scientific Council of the Biology and Soil Department, Irkutsk State University, Irkutsk, Russian Federation (30 November 2007, permission No. 3).

### 3.2. Laser Confocal Microscopy

#### 3.2.1. F-actin Staining

Actin microfilaments were stained with the bicyclic heptapeptide phalloidin conjugated with the Alexa Fluor 488 fluorescent label (Alexa Fluor™ 488 phalloidin, Thermo Fisher Scientific, Waltham, MA, USA, Cat. No. A12379) using the method detailed previously in [141]. The gill fragments were fixed in 4% paraformaldehyde (Merck, Burlington, MA, USA, Cat. No. P6148) prepared in PBS (pH = 7.4) for 15 min. Next, the samples were permeabilized in PBS with 0.3% Triton X100 (AppliChem GmbH, Darmstadt, Germany, Cat. No. 142314) for 30 min. After the permeabilization, the tissue fragments were stained with Alexa Fluor™ 488 phalloidin (165 nM solution) for 40 min. Next, nuclei in the samples were stained with Hoechst 33342 (2.0 µg/mL in PBS). After each phase of the sample preparation, the samples were washed three times in PBS.

#### 3.2.2. Programmed Cell Death Analysis

The intensity of programmed cell death processes in the gills was assessed by the terminal deoxynucleotidyl transferase-dUTP nick end labeling (TUNEL) method using the Click-iT™ Plus TUNEL Assay for in situ apoptosis detection with Alexa Fluor™ dyes (Thermo Fisher Scientific, Waltham, MA, USA, Cat. No. C10618) [39,141]. The material was fixed for 15 min in 3.7% paraformaldehyde. After washing three times in PBS, the gill fragments were permeabilized for 30 min in PBS with 0.3% Triton X100. Next, according to the Click-iT™ Plus TUNEL Assay Kit manufacturer’s instructions, the samples were washed three times with deionized water and built up at the 3′-OH ends of fragmented DNA with nucleotides (5-Ethynyl-2′-deoxyuridine 5′-triphosphate) using terminal deoxynucleotidyl transferase (TdT), with the subsequent conjugation of the inserted nucleotides with Alexa Fluor 594 picolyl azide dye as a result of the click reaction. After washing three times in PBS with 3% BSA (Santa Cruz Biotechnology, Inc., Dallas, USA, Cat. No. sc-2323), nuclei were stained with 2.0 µg/mL Hoechst 33342 solution (Thermo Fisher Scientific, Waltham, MA, USA, Cat. No. H3570) for 15 min. After washing the samples three times with PBS, the gill fragments were transferred to a glass slide, embedded in ProLong Gold Antifade Mountant (Thermo Fisher Scientific, Waltham, MA, USA, Cat. No. P36930), and sealed with a coverslip. For the positive control of TUNEL staining, we used DNAse I treatment, and for the negative control, tissue fragments were not treated with TdT, according to the Click-iT™ Plus TUNEL Assay Kit manufacturer’s protocol.

#### 3.2.3. Cell Proliferation Assay

The proliferative activity of gill cells was assessed by recording the incorporation of bromodeoxyuridine into the DNA of cells going through the S phase of the cell cycle [142]. To this end, we used the FITC BrdU Flow Kit (Becton Dickinson, Franklin Lakes, NJ, USA, Cat. No. 559619). Twelve hours before sampling, we injected 5 µL of BrdU solution (10 mg/mL, 32.5 mM in 1× DPBS) per 1 g of body weight into the abdominal cavity of the animal [85,143]. After autopsy, the detection of BrdU-positive cell nuclei in the gill tissue was performed according to the manufacturer’s instructions for the FITC BrdU Flow Kit. This process included fixation with the BD Cytofix/Cytoperm Buffer, permeabilization with the BD Cytoperm Permeabilization Buffer Plus, postfixation with the BD Cytofix/Cytoperm Buffer, treatment with DNase (diluted to 300 µg/mL in DPBS) to expose BrdU epitopes, and staining with FITC-conjugated anti-BrdU antibodies. After each stage in the sample preparation, the gill fragments were washed in BD Perm/Wash Buffer. Next, the tissue samples were stained with 7-AAD for 15 min to identify nuclei, transferred to a glass slide, and mounted in ProLong Gold Antifade Mountant.

#### 3.2.4. Microscopy and Image Analysis

Image acquisition was performed using an LSM 710 laser confocal microscope (Carl Zeiss, Oberkochen, Germany) in “stack” scan mode with “best signal” (slowest acquisition with configuration changes (laser/filter) between each image). For Alexa Fluor 488 phalloidin/Hoechst 33342-stained preparations, the following scanning settings were used: objective: Plan-Apochromat 63×/1.40 Oil DIC M27; filters: 410—490 nm; lasers: 1 track 405 nm: 4.0%; 2 track 488 nm: 4.0%. Slides stained with Alexa Fluor 594 picolyl azide dye/Hoechst 33342 were scanned with the following settings: objective: Plan-Apochromat 20×/0.8 M27; filters: 410—579 nm; lasers: 1 track 405 nm: 3.0%, 2 track 561 nm: 3.0%. The scan settings for the FITC-conjugated anti-BrdU antibodies/7-AAD-stained preparations were as follows: objective: Plan-Apochromat 20×/0.8 M27; filters: 1 channel: 500—550 nm, 2 channel: 610—691 nm; lasers: 1 track 561 nm: 4.0%, 2 track 488 nm: 5.0%. The analysis of the obtained Z-stacks, assessment of the quantitative indicators, and extraction/processing of the images were carried out using the following software: Zen 2010 (Carl Zeiss, Oberkochen, Germany), Imaris Viewer 9.6.0 and Imaris Bitplane 7.2.3 (Bitplane AG, Belfast, UK), and Adobe Photoshop CS6 extended (Adobe, Inc., San Jose, CA, USA). The number of TUNEL- and BrdU-positive nuclei in 1 × 10^6^ μm^3^ of tissue was assessed using Imaris Bitplane 7.2.3.

### 3.3. Statistical Analysis

Three groups of 10 animals each were used in the experiment. The data analysis was carried out in the Statistica 10 software environment (Stat Soft Inc., Tulsa, OK, USA) using nonparametric statistics to calculate the median and quartiles; intergroup differences were analyzed using the Kruskal–Wallis test. Intergroup differences were considered statistically significant at *p* ≤ 0.05.

## 4. Conclusions

Generally, soot exposure on days 5 and 14 induces forming zones with degenerative rearrangements of the actin cytoskeleton in the cells of fish gills: the dissociation of the F-actin framework of adhesion belts and actin patches, with actin aggregating to clumps or continuous perinuclear zones. In the areas of tissue with degenerative changes in the actin cytoskeleton, cell groups with adaptive thickening of the actin adhesion belts appeared. On day 14, newly formed migrating cells with special torpedo-shaped actin “sheaths” are observed. The exposure to soot microparticles promotes an apoptosis intensity increase in the gills on days 5 and 14 of the experiment. A decrease in proliferative activity of the gill cells on day 5 and an increase in cell proliferation on day 14 of soot exposure is shown. This paradoxical increase in cell proliferative activity in gills on day 14 under the impact of soot may be a compensatory process aimed at maintaining the necessary level of gill functions under the conditions of toxic soot exposure, and this process may occur until the gills’ recovery reserve is depleted. Together, these processes can cause dysfunction of the gills and affect the viability of fish. The obtained data will form the basis for the creation of a general conception of mechanisms of the toxic effect of soot microparticles on the cells of various organisms, as well as for the development of technologies for the bioindication of pollution and monitoring aquatic ecosystems.

## Data Availability

Not applicable.

## Supplementary Materials

The following supporting information can be downloaded at: https://www.mdpi.com/article/10.3390/ijms242015146/s1.
