# Supplementary material for: Degenerative and Regenerative Actin Cytoskeleton Rearrangements, Cell Death, and Paradoxical Proliferation in the Gills of Pearl Gourami (Trichogaster leerii) Exposed to Suspended Soot Microparticles
Source: Int J Mol Sci. 2023 Oct 13;24(20):15146. doi: 10.3390/ijms242015146 (PMC10607021; doi:10.3390/ijms242015146)
Supplement: Supplementary file 1 [file ijms-24-15146-s001.zip › Supplementary table 1.pdf]

Supplementary Table 1. Elemental composition and PAH content in soot microparticles used in experiment.

Method of analysis	Soot microparticle components	Content (%)
**Trace element energy-dispersive analysis (EDAX) data (elemental composition)**	carbon (C)	67
	oxygen (O)	14
	calcium (Ca)	4
	potassium (K)	3.8
	chlorine (Cl)	2.6
	sulfur (S)	2.3
	silica (Si)	1.9
	aluminum (Al)	1.63
	magnesium (Mg)	1.5
	manganese (Mn)	0.4
	sodium (Na)	0.3
**Gas chromatography–tandem mass spectrometry data (PAHs)**	phenanthrene	11
	fluoranthene	12
	pyrene	10
	benzo[*a*]anthracene	9
	chrysene	10
	benzofluoranthenes	9
	benzopyrenes	13
	indeno[*1,2,3-c,d*]pyrene	10
	benzo[*ghi*]perylene	7
