# Supplementary figures and images for: Degenerative and Regenerative Actin Cytoskeleton Rearrangements, Cell Death, and Paradoxical Proliferation in the Gills of Pearl Gourami (Trichogaster leerii) Exposed to Suspended Soot Microparticles
Source: Int J Mol Sci. 2023 Oct 13;24(20):15146. doi: 10.3390/ijms242015146 (PMC10607021; doi:10.3390/ijms242015146)

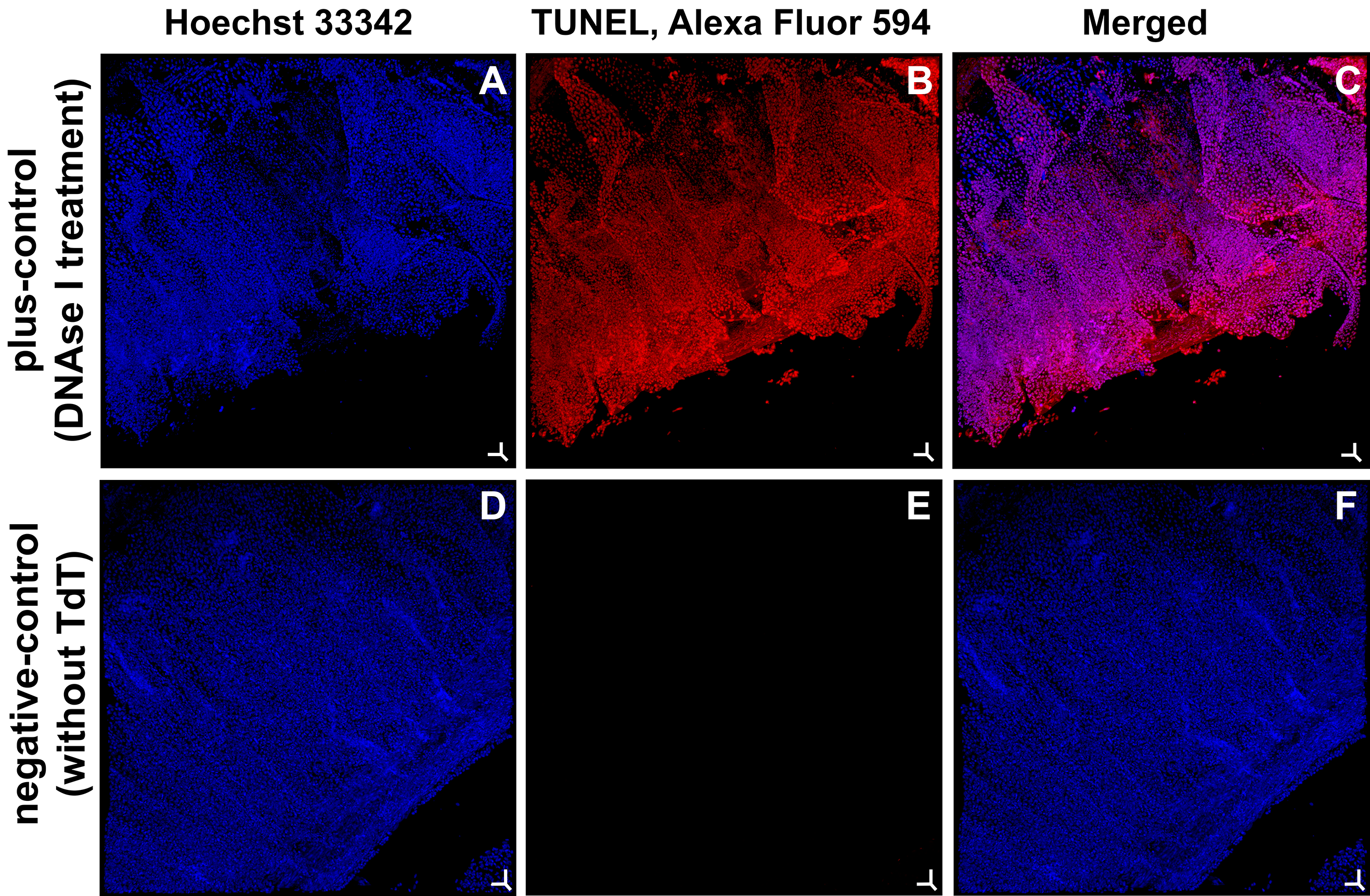

Supplement: Supplementary file 1 [file ijms-24-15146-s001.zip › Supplementary_figure_1.tif]

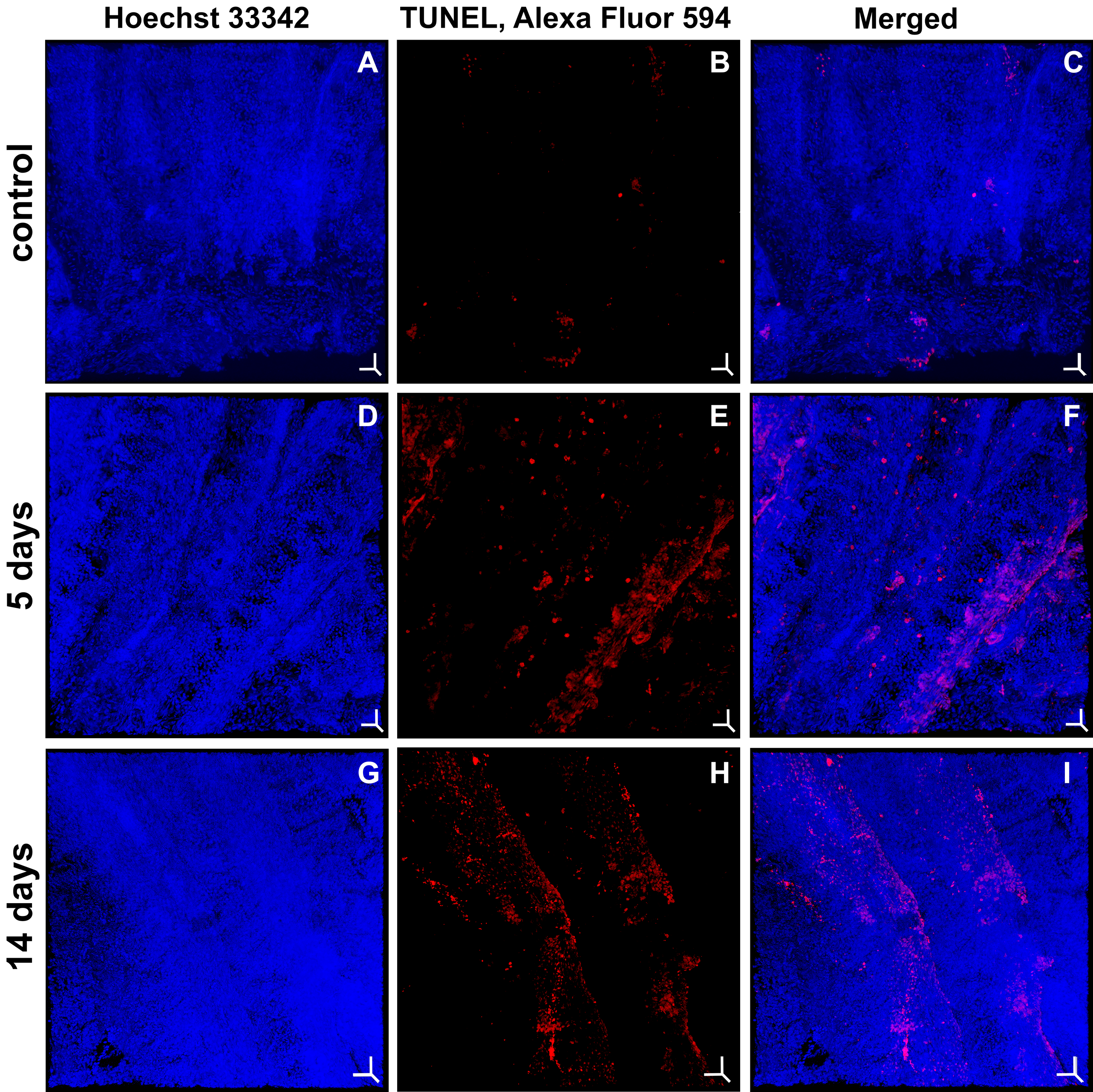

Supplement: Supplementary file 1 [file ijms-24-15146-s001.zip › Supplementary_figure_2.tif]

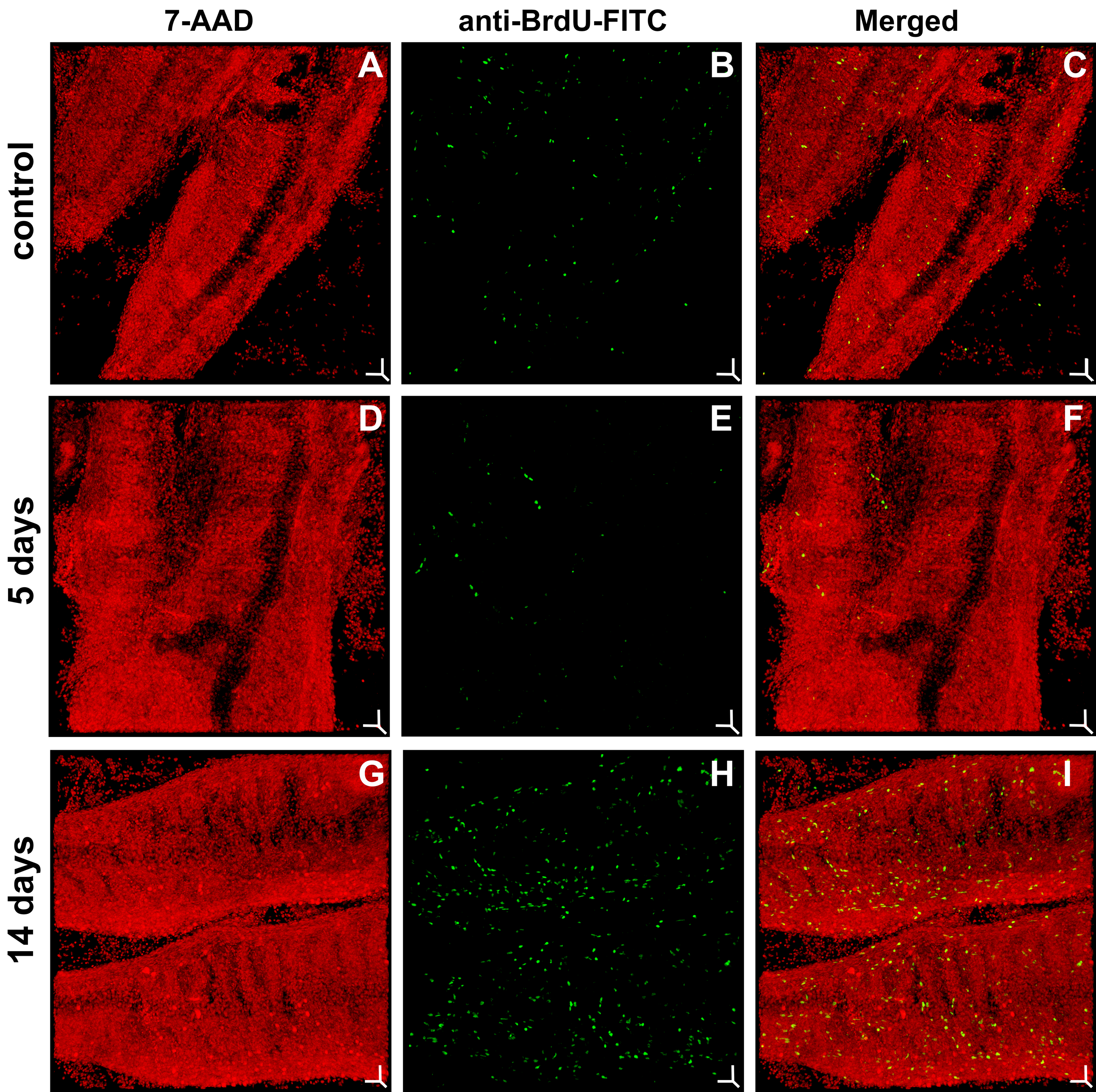

Supplement: Supplementary file 1 [file ijms-24-15146-s001.zip › Supplementary_figure_3.tif]
